# Human locomotion over obstacles reveals real-time prediction of energy expenditure for optimized decision-making

**DOI:** 10.1098/rspb.2023.0200

**Published:** 2023-06-14

**Authors:** Katherine A. J. Daniels, J. F. Burn

**Affiliations:** ^1^ Department of Sport and Exercise Sciences, Manchester Metropolitan University, Manchester M1 7EL, UK; ^2^ Queen's School of Engineering, University of Bristol, Bristol BS8 1TR, UK; ^3^ Manchester Metropolitan University Institute of Sport, Manchester M1 7EL, UK

**Keywords:** locomotion, optimization, human movement, biomechanics, energetics, manoeuvres

## Abstract

Despite decades of evidence revealing a multitude of ways in which animals are adapted to minimize the energy cost of locomotion, little is known about how energy expenditure shapes adaptive gait over complex terrain. Here, we show that the principle of energy optimality in human locomotion can be generalized to complex task-level locomotor behaviours requiring advance decision-making and anticipatory control. Participants completed a forced-choice locomotor task requiring them to choose between discrete multi-step obstacle negotiation strategies to cross a ‘hole’ in the ground. By modelling and analysing mechanical energy cost of transport for preferred and non-preferred manoeuvres over a wide range of obstacle dimensions, we showed that strategy selection was predicted by relative energy cost integrated across the complete multi-step task. Vision-based remote sensing was sufficient to select the strategy associated with the lowest prospective energy cost in advance of obstacle encounter, demonstrating the capacity for energetic optimization of locomotor behaviour in the absence of online proprioceptive or chemosensory feedback mechanisms. We highlight the integrative hierarchic optimizations that are required to facilitate energetically efficient locomotion over complex terrain and propose a new behavioural level linking mechanics, remote sensing and cognition that can be leveraged to explore locomotor control and decision-making.

## 1. Introduction

Understanding how the energy cost of locomotion shapes the way animals move is a long-standing fundamental challenge that spans the fields of physiology, ecology and neuroscience. Many aspects of legged locomotion appear to be optimized to minimize energy expenditure, including the freely chosen gaits [1–3], speeds [1,4], step frequencies [5–8] and step widths [9,10] of humans and other animals. More recently, it has been shown that the principle of energy cost minimization can also explain locomotor behaviours such as turning and changing speed in humans [11–13]. Together, these studies provide a solid foundation for understanding the energetic optimization of locomotion and predicting behaviour in simple environments where the ground surface is uniform, level and hard. In moving towards a unified understanding of locomotor optimization, it is necessary to extend the scope of investigation to consider energy cost minimization over natural terrain. The natural environment in which animals live and move is often characterized by uneven ground with multi-scale spatial variation in substrate geometry and material properties. The ground underfoot can hence change from step to step, necessitating a cascade of adaptations to steady-state gait. Animals have evolved a set of manoeuvres for negotiating such terrain, including leaping [14], changing direction [15], stepping onto raised/lowered ground [16], elevating the swing limb [17], and locally modifying step length and step width [18–21]. The energetic implications of these manoeuvres are considerable: human gross metabolic energy expenditure when walking over challenging rocky terrain can be more than twice that of walking over level ground [22], and even small variations (up to 2.5 cm) in terrain substrate height have been shown to increase energy expenditure by over 20% [23].

Humans appear to be remarkably sensitive to energy cost and will initiate gait modifications to achieve energy savings of less than 5% [24]. We hence might expect that, where additional locomotor degrees of freedom are introduced by the properties of natural terrain, the resulting movement choices would also be optimized to minimize energy expenditure. For example, if there are several possible routes or manoeuvres that can be implemented within a bout of locomotion we might anticipate that the chosen option would be the one associated with the lowest energetic cost. There is some evidence to support this hypothesis at both large and small spatiotemporal scales. Energy cost minimization appears to influence the selection of routes through geographic landscapes at large scale by naturally ranging and migrating terrestrial animals, although this optimization is understood to be largely reliant on existing spatial knowledge rather than direct sensing [25–28]. At a smaller scale, energy minimization seems to be a consideration when local visual information is used by humans for anticipatory control of limb trajectories and foot placements: in situations where an alternative foot placement location is required for a single step, the preferred option is often that which minimizes muscle activation or mechanical work requirements [29,30].

Despite the considerable proportion of the locomotor repertoire that appears to be explained by energetic optimization, it remains unknown whether terrestrial locomotion is energetically optimized at the extensive intermediate scale in which multi-step manoeuvres are planned and implemented. This scale captures many locomotor behaviours that are key to success in foraging, migration and predator–prey interaction, such as negotiating obstacles in the path of travel. For such manoeuvres, local behavioural decisions affecting multiple gait cycles with differing dynamics and energy costs must be made using visual information. For example, a fallen log in the path of travel might afford leaping over, walking over, climbing over, crawling under or deviating around. Each of these strategies requires adaptive control of multiple gait cycles with step-to-step variation in constraints, kinematics and energy costs. The preferred strategy must be selected and initiated in advance of obstacle contact, which requires visual information for feedforward control of movement—the animal cannot use online feedback to freely switch backwards and forwards between strategies during an obstacle negotiation manoeuvre because of the necessarily sequential nature of the movement elements comprising each strategy. Energetic optimization of locomotion at this scale would not represent a trivial extension to the capability that has been demonstrated to date. Instead, it would reveal the ability to integrate the prospective energy costs of potential locomotor behaviours across multiple heterogeneous steps, and to do so without access to online proprioceptive and metabolic feedback mechanisms. If evidenced, this would indicate that the extensive repertoire of multi-element locomotor manoeuvres and decisions observed in the real world are candidates for energy-based optimization, and demonstrate a new scale at which control principles, mechanisms and objective functions for non-steady-state locomotion can be explored.

An ideal task for probing this visually guided behavioural optimization of locomotion is the negotiation of locally elevated or lowered regions of terrain that can be crossed in just a few steps using a small number of discrete strategies. These regions might represent obstacles such as rocks, fallen logs, ditches and potholes. A cuboidal hole in the ground provokes locomotor behaviours demonstrating many common features of multi-step manoeuvres. It can be crossed using different basic strategies: by stepping across the gap from one side to the other or by stepping down into the base of the hole and then up the other side. Each potential strategy has implications for energetic cost due to the requirement to modify step length [8,31] or to lower and then raise the centre of mass (CoM) [32,33] during the manoeuvre. The chosen strategy must also be selected prior to the crossing, as the mechanics of the two manoeuvres diverge substantially in the initial step. Finally, the task is experimentally tractable because the energetic demands of crossing behaviour for both strategies can be easily manipulated by altering basic obstacle geometry (hole depth and length).

Here we first used previously published empirical data to model the energetic implications of choosing each of these two basic strategies to negotiate hole obstacles of varying geometry. We demonstrated that the energetically optimal strategy was dependent on both obstacle depth and obstacle length, and that this optimal strategy differed within the range of obstacle sizes comfortably traversable by walking humans. We then used experimental manipulation of obstacle length and depth to generate a cost surface for each manoeuvre, and tested the hypothesis that relative mechanical energy cost integrated across the multi-step obstacle negotiation task would predict preferred strategy in a two-alternative forced choice paradigm.

## 2. Material and methods

### (a) Initial modelling of relative energy cost for obstacle negotiation manoeuvres

**Figure 1.**
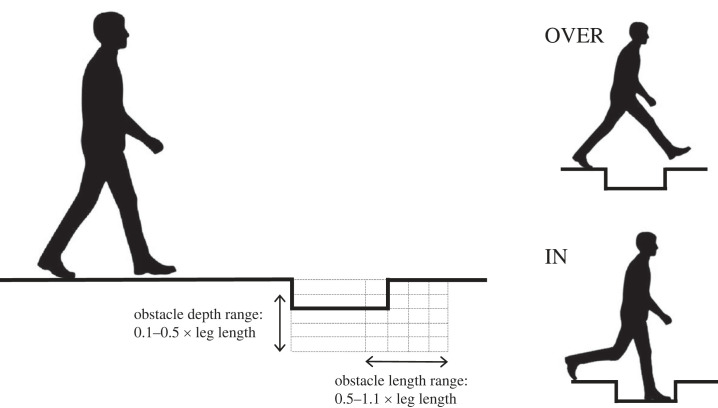
Experimental configuration. In the first part of the study, participants crossed a lowered region of ground (obstacle) using their preferred traversal strategy. The length and depth of the obstacle was manipulated prior to each trial. In the second part of the study, the non-preferred strategy was used instead for each presented obstacle geometry.

When a hole in the ground of substantial width is encountered during walking, there are two discrete strategies that can be used to traverse the obstacle without re-routing (figure 1): (i) stepping directly over the hole from one side to the other (OVER strategy) and (ii) stepping down into the base of the hole and then back up the other side (IN strategy). A decision must thus be made between these two possibilities if the obstacle is to be successfully traversed. For the decision to represent a valid indication of strategy preference, rather than merely perceived capability, it is necessary that both alternatives are perceived as viable by the participants. A range of obstacle length–depth combinations comfortably traversable by able-bodied young adults using both the OVER and the IN strategies was thus established during pilot work, spanning obstacle depths of 10–50% leg length and obstacle lengths of 50–110% leg length.

A second requirement, if energy cost minimization is to be robustly tested as a candidate objective function, is that the energy cost of the task must differ between the two strategies and the identity of the lowest-cost strategy should change across the range of length–depth combinations explored. We thus first modelled the estimated metabolic cost of the IN and the OVER strategies across our identified range of viable hole geometries based on previously published metabolic energy cost functions. For this purpose, metabolic cost of transport for the OVER strategy was estimated from the previously measured cost of walking with an extended step length [8], with the assumption that step length would be extended by the minimum amount required to span the obstacle without altering walking speed. The metabolic cost of both stepping up and stepping down between height levels is approximately proportional to the change in height level, and the cost of the combined movement is dominated by the cost of the upward step as mechanical work is done to raise the CoM [32]. Metabolic cost of transport for the IN strategy was hence estimated from the sum of the energy costs of stepping down and stepping up at the designated obstacle height, using the empirically derived equations presented in the literature [32] (see electronic supplementary material, Methods, for further details).

### (b) Experimental participants

Thirteen participants took part in the experimental study (eight female and five male, age range 19–33 years, mean body mass 72.3 ± s.d. = 15.5 kg, mean leg length 0.88 ± s.d. = 0.04 m). All were free of self-reported musculoskeletal, visual and neurological impairments. The protocol was approved by the faculty Human Research Ethics Committee and participants gave informed written consent.

### (c) Experimental apparatus

Participants walked along a 14.2 m × 0.6 m trackway that was flat, level and straight, raised 0.48 m above the ground. A metre-long section of the trackway, located 7.2 m from the start, was comprised of multiple rigid polystyrene infill sections (SP-X Styrofoam, The Dow Chemical Company, Midland, USA) that could be removed to create a cuboidal ‘hole’ (the *obstacle*) spanning the full width of the track and with manipulable depth and length (figure 1).

Twenty-five different obstacle length–depth combinations were generated for each participant. Target obstacle dimensions were defined relative to leg length (standing greater trochanter height, *l*), with five obstacle depths of 0.1*l*–0.5*l* at intervals of 0.1*l* and five obstacle lengths of 0.5*l*–1.1*l* at intervals of 0.15*l*. The maximum deviation in constructed obstacle size from the calculated target value was 25 mm (length) and 26 mm (depth), resulting from the spatial resolution of the infill sections.

### (d) Preparation of participants

Passive retroreflective spherical markers of 14 mm diameter (Qualisys AB, Gothenburg, Sweden) were attached on the left and right side of the body using double-sided adhesive tape over the following anatomical landmarks: head of the fifth metatarsal, lateral malleolus, lateral femoral condyle, greater trochanter, acromioclavicular joint, lateral epicondyle of the humerus, dorsal tubercle of the radius. Tight-fitting clothing was worn to minimize artefactual marker movement and the experiment was performed with bare feet.

### (e) Experimental procedure

The experimental protocol was divided into two parts: the first in which the participant completed the obstacle crossing task using their freely selected preferred traversal strategy and the second in which they were instructed to use their non-preferred strategy. This enabled direct comparison of the energy and time costs of preferred and non-preferred strategies at each of the 25 obstacle length–depth geometry combinations presented.

Participants began every trial standing at the start of the track. They then walked at a self-selected comfortable speed from one end of the track to the other while three-dimensional marker trajectories were recorded at 120 frames per second by a three-dimensional optical motion capture system (Oqus 300 with Qualisys Track Manager, Qualisys AB, Gothenburg, Sweden), and then returned to the starting position via an alternative route until instructed to perform the next trial. To minimize the likelihood of a systematic relationship between the position of the obstacle and the spatial kinematics of the stride cycle at the time of encounter, participants were randomly allocated to one of four position markers placed 0.3 m apart at the start of the track for each trial. They were instructed to place the toe of either the right or the left foot level with the named position marker and to use that as a starting position for the trial, protracting the contralateral leg to initiate walking.

Four familiarization trials were performed before data collection commenced, in which the participant traversed the largest obstacle (depth and length) to be encountered during the study by both stepping down into it and by stepping over it. It was thus confirmed that the participant was able to use both strategies for traversal, and was comfortable doing so, even at the maximum obstacle dimensions to be tested.

For the first set of data collection trials, participants were instructed to imagine each trial as a short section of a longer walk, such as would be taken on a journey through the real-world environment during normal daily life. They were instructed to attain a comfortable walking speed at the beginning of the trial then to make any adjustments to gait that they would naturally in order to cross the gap and to return to a comfortable walking pace by the end of the track. Twenty-five trials were collected, in each of which the participant traversed an obstacle comprising one of the assigned length–depth combinations. All possible combinations were presented in a random order, and it was recorded after each trial whether the participant had traversed the obstacle by stepping down into the base of the obstacle and then back up the other side (the IN traversal strategy) or by stepping over the gap (the OVER traversal strategy).

After the completion of this first set of trials, the IN and OVER strategies were described to the participant. They were told that in the next stage of the experiment they would be instructed before the start of each trial which of these two strategies they were required to use, and that they were to abort the trial and inform the experimenter if they were presented with any obstacle they believed they were unable to comfortably traverse using the required strategy. The participant then performed a second set of trials, in which they were required to traverse the obstacle using the strategy they had not selected in the previous part of the experiment, i.e. if they had previously chosen to traverse an obstacle of given dimensions using the IN strategy, they were now asked to traverse it using the OVER strategy. The participant was advised that they were permitted to take one or more steps in the base of the obstacle during IN traversal strategy trials if they wished to do so. As no differences were expected in OVER traversal strategy mechanics across this relatively small range of depths [34], participants who had chosen to use the OVER strategy to traverse one of the depths at a given length were not asked to perform OVER strategy trials for other depths at that same length. Those who had not chosen to use the OVER strategy to traverse any depths at a given length were instructed to do so at only one depth (0.5*l*).

The majority of IN strategy traversals involved the placement of a single foot in the base of the hole followed by a step up back onto the raised track. Four participants, however, took a complete step in the base of the hole (two consecutive foot placements) during at least one preferred strategy trial, and six participants did the same during at least one non-preferred strategy trial. These additional steps were considered part of the traversal manoeuvre and included in the mechanical work and speed calculations for the trial.

### (f) Data processing

A total of 580 trials were recorded from 13 participants. The data from six trials were discarded due to markers dropping out of camera view so the data subsequently analysed consisted of motion capture data for each of the remaining 574 trials. Marker trajectory data were filtered using a fourth-order zero phase shift Butterworth low-pass filter with a corner frequency of 6 Hz. The start and end of each stance phase was identified automatically using an algorithm incorporating horizontal velocity of the metatarsal and malleolus markers and verified by visual inspection (see electronic supplementary material, Methods, for details). A *step* was defined as the period from the start of a stance phase to the ultimate frame before the start of the subsequent stance phase of the contralateral leg.

Metabolic cost of transport cannot be measured directly for transient manoeuvres because of the complicated and temporally delayed relationship between instantaneous energy cost and respiratory gas composition [35]. Total mechanical cost of transport (CoT_tot_) was therefore the main outcome measure for the experimental study, representing the mechanical work performed to move the body. CoM mechanical cost of transport (CoT_CoM_; the work per unit distance associated with body CoM movement) was also calculated and analysed separately. To allow these metrics to be calculated, an 11-segment model of the participant was constructed using three-dimensional position data from the 14 markers (head and trunk, upper arms, forearms, thighs, shanks, feet). These kinematic data were combined with published anthropometric values for segment relative masses and mass distributions [36] to calculate the position of the CoM for each frame. The instantaneous mechanical energy of the CoM was calculated as the sum of kinetic and gravitational potential energy, and CoM mechanical work was then calculated as the sum of CoM mechanical energy increments with respect to time [37,38]. Total (sum of CoM and internal) mechanical work was calculated as the sum of increments in segmental mechanical energies, assuming within-limb but not between-limb exchanges between kinetic and gravitational potential energy [39,40].

Both total and CoM mechanical work were calculated for all individual steps comprising the complete obstacle traversal task. For IN strategy traversals, this was all steps from the initial lead leg step down into the base of the obstacle to the trail leg step back up onto the raised trackway (inclusive). For OVER strategy traversals, this was the lead and trail leg steps over the gap. Mass-specific CoT_tot_ and CoT_CoM_ were computed for each step and for the complete traversal (all steps comprising the traversal task) by expressing total and CoM mechanical work respectively per kilogram body mass and unit distance. *Traversal speed* was computed by dividing the sum of step lengths by the sum of step times for the complete traversal, and expressed as a Froude number (dimensionless speed) calculated as $v{\left(\sqrt{gl}\right)}^{-1}$, where *v* = step speed in m s^−1^, *g* = acceleration due to gravity (9.81 m s^−2^) and *l* = leg length in m. The step located three steps prior to the first obstacle traversal step was defined as the *approach step*, and its speed (*approach speed*) was calculated by dividing step length by step time and non-dimensionalizing as above.

### (g) Analysis

Four candidate control targets for strategy selection were quantitively tested for consistency with the observed locomotor behaviour: minimization of CoT_tot_ for the traversal task, minimization of CoT_tot_ of the highest-cost individual step within the task, maximization of traversal speed and conservation of locomotion speed. The latter was quantified as the absolute difference between approach speed and traversal speed. Minimization of task CoT_tot_ defines the energetically optimal strategy for the traversal, whilst minimization of step CoT_tot_ would represent a locally optimal solution. Maximization of forward speed has been found to contribute to the selection of foot placements when moving over cluttered terrain [20,41], and conservation of walking speed would minimize the influence of the obstacle on the time taken to complete the journey. CoT_tot_ and CoT_CoM_ were expected to be highly correlated for this task, but minimizing CoT_CoM_ for the traversal task and for the highest-cost step were nevertheless also analysed as potential control targets for the sake of completeness.

For each candidate control target variable, a logistic regression model [42] was constructed to model the relationship between the magnitude of the advantage in selecting the OVER strategy and the observed probability of this strategy being preferentially selected by the participants, deriving an equation of the form

2.1 $${\mathrm{LO}}_{\mathrm{OVER}}=\ln\frac{\;p}{1-p}=b_{0}+b_{1}x,$$

where LO_OVER_ is the log odds of the OVER strategy being selected, *b*_0_ and *b*_1_ are the model parameters, *p* is the binomial proportion for OVER strategy selection and *x* is the absolute advantage obtained by selecting the OVER strategy.

The direction of the advantage was defined as positive if the OVER strategy was associated with the lowest cost of transport (candidate variables: traversal task CoT_tot_ and CoT_CoM_; single-step CoT_tot_ and CoT_CoM_), highest speed (candidate variable: traversal speed maximization) or speed closest to the approach speed (candidate variable: locomotion speed conservation). The magnitude of the advantage was defined as the absolute difference between the mean values of the candidate variable for the two strategies. For example, if mean task CoT_tot_ when traversing an obstacle of given dimensions was 2 J kg^−1^ m^−1^ for the OVER strategy and 3 J kg^−1^ m^−1^ for the IN strategy, the advantage for selecting the OVER strategy based on task CoT_tot_ would be 1 J kg^−1^ m^−1^.

The models were then used to evaluate behavioural decisions for consistency with each candidate control target by analysing each fitted model for two features: (i) a significant positive *b*_1_ coefficient, indicating that an increase in the advantage (based on the relevant candidate variable) of choosing the OVER strategy was associated with an increase in the odds of choosing the OVER strategy; and (ii) no significant *b*_0_ coefficient, indicating that no bias was identified in the probability of selecting a given strategy when there was no advantage present for either (i.e. the 95% confidence interval for the log odds of selecting a given strategy was inclusive of zero when both strategies would result in the same value of the control target variable). A model with these features would be consistent with a control principle in which participants were equally likely to choose the IN and OVER strategies when there was no advantage to selecting one or the other, and increasingly likely to choose a given strategy as the advantage incurred by doing so increased.

## 3. Results

### (a) Empirical metabolic cost functions predict a transition in energetically optimal traversal strategy within the range of comfortably traversable obstacle dimensions

**Figure 2.**
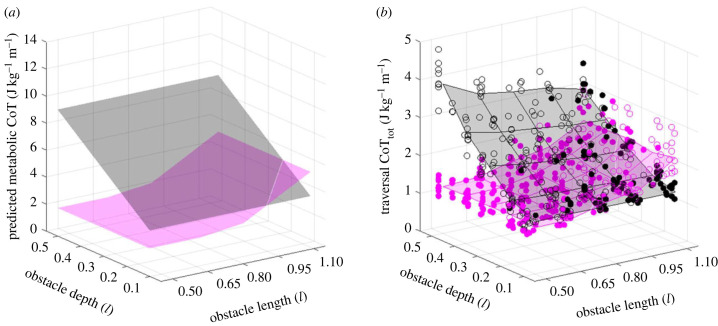
Predicted metabolic cost of transport (*a*) and calculated total mechanical cost of transport (*b*) for the complete obstacle traversal task, displayed for OVER strategy traversals (pink) and IN strategy traversals (dark grey). Obstacle length and depth reported as proportion of participant leg length (*l*). Predicted values in (*a*) based on the empirical metabolic costs for extended walk step lengths and step heights reported in [8,32]. Coloured surfaces in (*b*) generated using polynomial regression models. Circles in (*b*) present mechanical cost of transport (CoT_tot_) values for all individual experimental trials: freely selected IN strategy (filled black circles), freely selected OVER strategy (filled pink circles), non-preferred IN strategy (open black circles) and non-preferred OVER strategy (open pink circles).

Obstacle geometry was expected to influence the energy cost of both the OVER and the IN strategies due to the increased energy cost associated both with increasing step length [8,31] and with elevating the CoM [32,33]. We therefore first demonstrated that the energy cost landscapes for the two possible behavioural strategies would be predicted to intersect within a range of obstacle lengths and depths comfortably traversable using both strategy options by modelling estimated energy costs using published metabolic cost data. Results are shown in figure 2*a*: the metabolic energy cost planes for the two strategies intersect within the evaluated range of obstacle geometries, indicating that a transition in preferred strategy from IN to OVER would be predicted with increasing obstacle length and decreasing depth if energy optimization were a control target for strategy selection decision-making.

### (b) The energetically optimal traversal strategy is dependent on both obstacle length and obstacle depth

The mechanical cost of transport for obstacle negotiation in our study was affected by the dimensions of the obstacle and by the strategy implemented, as predicted by the metabolic cost estimates. The IN strategy CoT_tot_ was relatively independent of obstacle length but increased with obstacle depth, whilst the OVER strategy CoT_tot_ increased with obstacle length (figure 2*b*). The CoT planes for the two strategies intersected, indicating that the optimal strategy based on mechanical energy cost also differed across the investigated range of obstacle length–depth combinations: CoT (both total and CoM; data for CoT_CoM_ presented in electronic supplementary material, figure S1) was lower for the IN strategy than for the OVER strategy when the obstacle was long and shallow, and lower for the OVER strategy than the IN strategy when the obstacle was short and deep.

**Figure 3.**
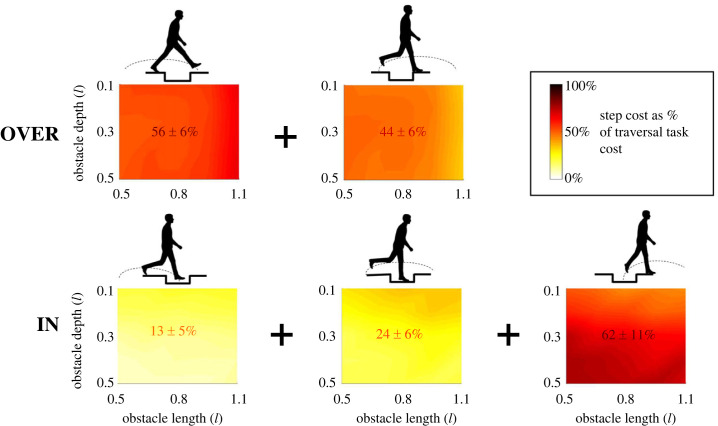
Contribution of each traversal step to the total mechanical energy cost for the task. Colour bar indicates the proportion of task mechanical work done in the relevant step for obstacles of a given length (horizontal axis) and depth (vertical axis). Mean ± s.d. contribution percentage across all obstacle geometries indicated numerically in the panel centre.

The mechanical energy cost of a complete traversal task can be broken down into the individual steps that comprise the task. The CoT_tot_ of the IN strategy was dominated by the cost of the step back up onto the trackway (62 ± 11% of traversal CoT_tot_ averaged across all obstacle depth–length combinations, up to 74 ± 3% at the greatest obstacle depths, compared with 13 ± 5% and 24 ± 6% for the step down into the base and its following step, respectively; figure 3), whereas the CoT_tot_ of the OVER strategy was more evenly weighted between the lead leg crossing step (56 ± 6%) and the trail leg crossing step (44 ± 6%). CoT_CoM_ was strongly correlated with CoT_tot_, both for the complete task (IN strategy *r* = 0.98, *p* < 0.001; OVER strategy *r* = 0.92, *p* < 0.001) and for individual steps (IN strategy *r* = 0.99, *p* < 0.001; OVER strategy *r* = 0.94, *p* < 0.001).

### (c) Anticipatory control guides strategy selection

**Figure 4.**
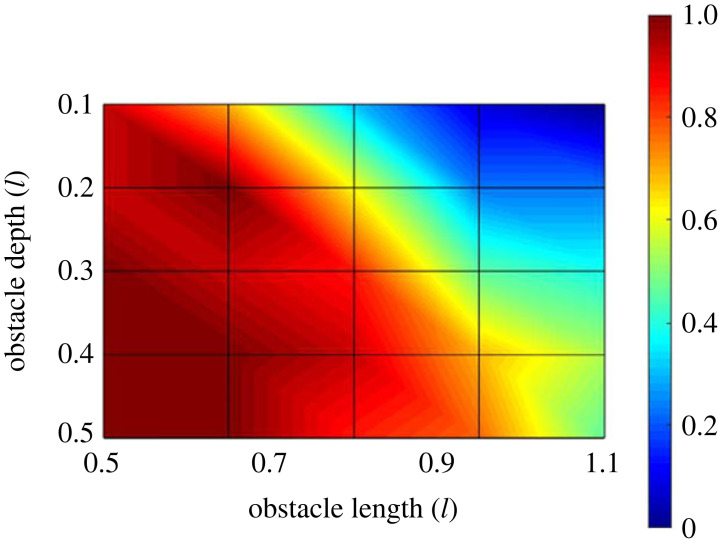
The effect of obstacle dimensions on obstacle traversal strategy selection. The proportion of free choice trials at each combination of relative obstacle dimensions for which the OVER strategy traversal was selected. Short, deep obstacles were preferentially traversed using this strategy (red region), whereas shallow, long obstacles were preferentially traversed using the IN strategy (blue region).

Participants were comfortable traversing all presented obstacles using both the IN and the OVER strategies, with a single exception in which one participant chose to abort the enforced trial at the largest obstacle length and depth. For obstacles presented during the first part of the experiment a decision was thus made between more than one viable traversal strategy for each trial. This decision was a function of *both* obstacle length and obstacle depth: the OVER strategy was preferentially selected to negotiate short, deep obstacles and the IN strategy to negotiate long, shallow obstacles (figure 4).

### (d) Strategy selection was consistent with behavioural optimization of energetic cost of transport for the locomotor task

**Figure 5.**
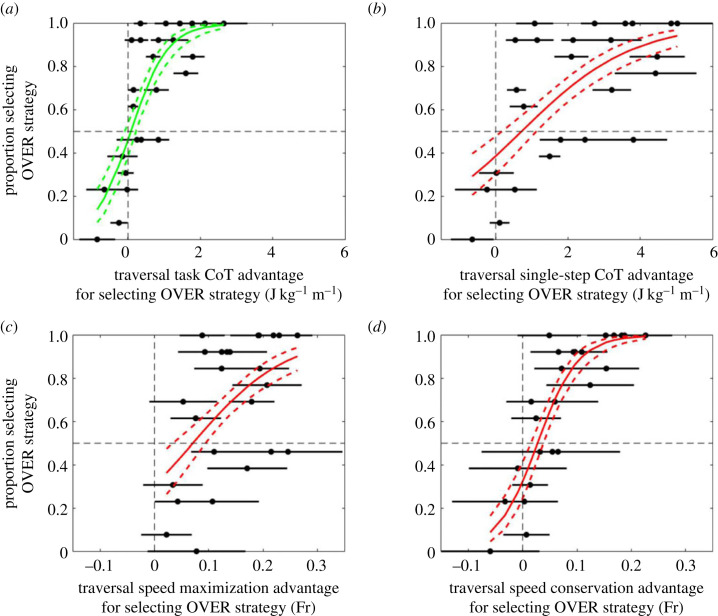
Relationship between obstacle traversal strategy relative cost and selection probability, based on objective functions (*a*) minimization of task-level CoT_tot_, (*b*) minimization of step-level CoT_tot_, (*c*) maximization of locomotion speed, and (*d*) conservation of locomotion speed. Fr = Froude number (dimensionless speed). Solid lines represent fitted logistic curves and dotted lines the corresponding 95% confidence interval. Each point (with associated horizontal bars) represents the mean ± s.d. difference in the outcome variable between the two traversal strategies for a single obstacle length–depth combination. The vertical dashed line at *x* = 0 indicates equal cost for IN and OVER strategy traversals: points to the left of this line represent lower cost for IN and points to the right of this line represent lower cost for OVER. The point of subjective equality (equal likelihood of selecting the IN and the OVER strategy) is marked by a horizontal line at *y* = 0.5.

Traversal strategy selection was consistent with task CoT_tot_ minimization but not with individual step CoT_tot_ minimization, locomotion speed maximization or locomotion speed conservation (figure 5 and table 1). For each individual obstacle length–depth combination, the magnitude of the task CoT_tot_ difference between the two possible strategies predicted the probability that a given strategy would be chosen (change in log odds for each 1 J kg^−1^ m^−1^ cost advantage increase = 1.97). Obstacles for which the task CoT_tot_ advantage of selecting a particular strategy was greatest were thus most likely to be traversed using that strategy, and obstacles for which the task CoT_tot_ of the two strategies was similar showed a weaker bias towards a consistent strategy preference (figure 5*a*). Only this candidate control target demonstrated both necessary logistic model features (no significant *b*_0_ coefficient; significant positive *b*_1_ coefficient)—the former requirement, representing unbiased strategy preference when the costs of the two strategies were equal, was not met for any of the other three investigated candidates (figure 5*b–d* and table 1). CoT_CoM_ results mirrored those of CoT_tot_ for the task and for the individual steps, as would be expected due to their close correlation, and are presented in the electronic supplementary material (figure S2).

**Table 1.** Logistic model coefficients and test statistics for each evaluated candidate control target. CoT_tot_ = total mechanical cost of transport.

candidate control target	*b*_0_	*b*_0_ *t*	*b*_0_ *p*	*b*_1_	*b*_1_ *t*	*b*_1_ *p*
task-level CoT_tot_	−0.16	−1.00	0.319	1.97	7.70	<0.001
step-level CoT_tot_	−0.46	−2.43	0.015	0.65	7.09	<0.001
speed maximization	−0.82	−2.99	0.003	11.57	5.90	<0.001
speed conservation	−0.74	−3.70	<0.001	26.86	8.14	<0.001

## 4. Discussion

Our findings demonstrate that the principle of energy optimality in human locomotion can be generalized to complex locomotor behaviours requiring anticipatory control and decision-making. When forced to choose between alternative multi-step manoeuvres to negotiate an obstacle in the path of travel, participants made decisions that minimized mechanical energy cost of transport for the complete obstacle crossing task. In doing so, they revealed the capacity to integrate predicted cost across multiple steps based on visual information and to use this information to select optimal movement sequences.

This study provides the first clear evidence in any animal species for energetically optimized locomotor decision-making in the absence of online proprioceptive or chemosensory feedback mechanisms. Unlike the optimization of continuous gait parameters in walking and running, it is not possible for online control to have contributed to movement strategy selection in this task. The nature of the two possible strategies meant that kinematics necessarily diverged at the onset of obstacle negotiation, so participants were obliged to commit to their chosen manoeuvre during the approach phase. By recording the preferred strategy for each obstacle length–depth combination on the first encounter, we eliminated the possibility of participants using direct feedback during exploration of the two alternatives. There was no significant difference in the proportion of participants selecting the energetically optimal strategy in their first trial and in their last trial of the experiment (*χ*^2^ = 0.87, *p* = 0.35), suggesting that sensorimotor exploration within the data collection session was also unlikely to be responsible for the observed optimization. This is in striking contrast to the extensive exploration period that appears to be required by most humans for energetic optimization of continuous gait parameters in novel contexts [43–45]. Instead, visual information obtained during the approach phase appears to have been used in a feedforward manner to guide decision-making based on prospective energy cost. This mechanism has been previously proposed to explain speed adjustments during walking on uneven terrain [46] and jumping kinematics in dogs [47].

The nature of the task not only necessitated strategy selection in advance of obstacle encounter but also required the energetic implications of multiple steps with different dynamics and mechanical work requirements to be incorporated into the decision. This indicates the capability to predict energy cost for adaptive gait and to integrate these cost predictions over multiple heterogeneous steps to plan and select the strategy with the lowest overall energy cost. The principle of expending additional energy in some phases of a movement to reduce overall movement cost is a known feature of locomotion at the scale of the individual gait cycle. For example, expending energy on an active ankle push-off in late stance can reduce the overall energy cost of walking by reducing work requirements during other phases of the gait cycle [48,49]. However, the demonstration of this capability at the scale of manoeuvre selection demonstrates a far more complex ability to plan multi-step adaptive gait sequences and optimize behaviour to minimize anticipated energy costs.

Our findings are equivocal regarding the neural mechanisms involved in optimizing obstacle negotiation behaviour. Visually sensed information specifying both the length and the depth of the obstacle must have been incorporated into the decision because the energetically optimal strategy was a function of both dimensions, but our results do not establish the perceptual or computational mechanisms responsible. Whilst it is highly unlikely that the participants had prior experience of the specific experimental task, it is feasible that longer-term feedback over evolutionary and ontogenetic timescales could enable mapping between the visual properties of an obstacle or obstacle component and its manoeuvre-specific energy costs. We speculate that this may inform an internal model for locomotor decision-making in complex environments that incorporates feedforward planning over multiple gait cycles to minimize energetic cost. While the concept of a planning horizon for optimization is commonly used in algorithms for predictive modelling and control of robotic systems (e.g. [50–53]), there has previously been little evidence that humans can model the energetic implications of upcoming adaptive gait cycles in complex novel environments. Incorporation of energy-based feedforward modelling over biologically plausible scales would be expected to improve predictions of human locomotor behaviour in contexts as diverse as urban design and military strategy, and to inform control principles for efficient bioinspired autonomous legged systems. The manoeuvres implemented within this study incorporated up to four steps of human–obstacle interaction, providing a lower bound for the planning horizon of such a model for this task.

We used a simple analytical approach to evaluate individual candidate control targets, testing a hypothesis that was based on the previously reported high prioritization of energy optimization in other locomotor contexts. However, locomotion is inevitably subject to multiple concurrent control priorities and constraints, such as the additional requirement to maintain stability during manoeuvres [15,54,55]. We thus evaluate energy-based control with the implicit understanding that the additional costs and trade-offs required to maintain stable locomotion are already accounted for within our measurements, rather than attempting to isolate an energy cost for the manoeuvre in the absence of any real-world constraints. We also acknowledge that any selection pressure acting to minimize the energetic cost of locomotion would be expected to operate on metabolic energy expenditure rather than on mechanical work. Several different approaches have been used to estimate the mechanical work of locomotion, each of which attempts to account for energy use based on a set of assumptions. The method we use here accounts for the changes in mechanical energy of the CoM and with the movements of the limbs relative to the CoM during locomotion by measuring positive increments in segmental mechanical energies, assuming that within-limb but not between-limb passive energy exchanges occur [39,40]. This approach does not account for elastic strain energy exchanges, antagonistic muscle co-contraction or simultaneous positive and ‘negative’ work being done on the CoM by the two legs during double-support phase. Some of these assumptions would be expected to lead to underestimates of the mechanical work done by muscle and some to overestimates, so the overall error in the model is dependent on the relative contributions of these components for any individual movement task. ‘Collisional’ models, which aim to account for the cost of redirecting the CoM upwards in each stance phase [56], offer an alternative approach and have been used successfully to account for the cost of continuous walking with different step lengths and widths [57]. The close agreement between figure 2*a* and *b* and between CoT_tot_ and CoT_CoM_ suggests that variation in mechanical work to change the energy of the CoM, as quantified by our method, either is, or is strongly correlated with, the greatest contributor to metabolic cost variation for this task. It may be that the control target is instead a correlate of energy, such as muscle activation or perceived effort, but energy minimization is a parsimonious explanation for the observed behaviour.

Spatial variations in terrain geometry and surface mechanical properties that affect local energy expenditure are common in both natural and built environments, so energy cost variation is a prevalent feature of locomotion in the real world. Although a single obstacle negotiation manoeuvre is a transient task, the energetic implications of strategy selection decisions may thus be substantial when all such manoeuvres across a journey, day or lifespan are considered—the cost of traversing the presented obstacles differed by up to 400% depending on which strategy was implemented. Ecological ‘energy landscapes’ have previously been constructed to relate the geographical location of an animal to its cost of transport, and thus to understand how migratory and foraging routes are influenced by energetic cost [26,27,58]. However, the strategy-dependency of obstacle negotiation cost shown by our results highlights the need to consider behavioural decisions at all scales when generating such models.

In conclusion, we have shown that the principle of energy optimality in human locomotion extends beyond steady-state gait and uniform terrain substrates to encompass task-level predictive control and selection of multi-step manoeuvres. The behavioural decisions made by our participants reveal the capability to (i) obtain information about the energetic implications of upcoming terrain using visual sensing, (ii) integrate prospective energy requirements for different discrete manoeuvres over multiple heterogeneous gait cycles, and (iii) use this information to drive locomotor decision-making. Our findings unify previous observations of energy optimality at higher and lower hierarchic levels with a new behavioural level linking mechanics, remote sensing and cognition. This intermediate level can be leveraged to explore both the optimizations required for economical movement over complex terrain and the mechanisms underlying sensorimotor control of legged locomotion. More generally, we conjecture that animals have adapted to minimize energy expenditure for locomotion at all scales where degrees of freedom to do so can be exploited.

## Data Availability

The data are provided in electronic supplementary material [59].

## References

[1] Hoyt DF, Taylor CR. 1981 Gait and the energetics of locomotion in horses. Nature **292**, 239-240. (10.1038/292239a0)

[2] Alexander RM. 1984 The gaits of bipedal and quadrupedal animals. Int. J. Rob. Res. **3**, 49-59. (10.1177/027836498400300205)

[3] Griffin TM, Kram R, Wickler SJ, Hoyt DF. 2004 Biomechanical and energetic determinants of the walk-trot transition in horses. J. Exp. Biol. **207**, 4215-4223. (10.1242/jeb.01277) 15531642

[4] Ralston HJ. 1958 Energy-speed relation and optimal speed during level walking. Int. Z. Angew. Physiol. **17**, 277-283. 13610523 10.1007/BF00698754

[5] Zarrugh MY, Radcliffe CW. 1978 Predicting metabolic cost of level walking. Eur. J. Appl. Physiol. Occup. Physiol. **38**, 215-223. (10.1007/BF00430080) 648512

[6] Cavagna GA, Franzetti P. 1986 The determinants of the step frequency in walking in humans. J. Physiol. **373**, 235-242. (10.1113/jphysiol.1986.sp016044) 3746673 PMC1182534

[7] Minetti AE. 1995 Optimum gradient of mountain paths. J. Appl. Physiol. **79**, 1698-1703. (10.1152/jappl.1995.79.5.1698) 8594031

[8] Bertram JEA. 2005 Constrained optimization in human walking: cost minimization and gait plasticity. J. Exp. Biol. **208**, 979-991. (10.1242/jeb.01498) 15767300

[9] Donelan JM, Kram R, Kuo AD. 2001 Mechanical and metabolic determinants of the preferred step width in human walking. Proc. R. Soc. Lond. B **268**, 1985-1992. (10.1098/rspb.2001.1761) PMC1088839 11571044

[10] Abram SJ, Selinger JC, Donelan JM. 2019 Energy optimization is a major objective in the real-time control of step width in human walking. J. Biomech. **91**, 85-91. (10.1016/j.jbiomech.2019.05.010) 31151794

[11] Brown GL, Seethapathi N, Srinivasan M. 2021 A unified energy-optimality criterion predicts human navigation paths and speeds. Proc. Natl Acad. Sci. USA **118**, e2020327118. (10.1073/pnas.2020327118) 34266945 PMC8307777

[12] Seethapathi N, Srinivasan M. 2015 The metabolic cost of changing walking speeds is significant, implies lower optimal speeds for shorter distances, and increases daily energy estimates. Biol. Lett. **11**, 20150486. (10.1098/rsbl.2015.0486) 26382072 PMC4614425

[13] Long LL, Srinivasan M. 2013 Walking, running, and resting under time, distance, and average speed constraints: optimality of walk–run–rest mixtures. J. R. Soc. Interface **10**, 20120980. (10.1098/rsif.2012.0980) 23365192 PMC3627106

[14] Daniels KAJ, Burn JF. 2021 Visuomotor control of leaping over a raised obstacle is sensitive to small baseline displacements. R. Soc. Open Sci. **8**, 201877. (10.1098/rsos.201877) 33959347 PMC8074954

[15] Jindrich DL, Qiao M. 2009 Maneuvers during legged locomotion. Chaos **19**, 026105. (10.1063/1.3143031) 19566265

[16] Grimmer S, Ernst M, Günther M, Blickhan R. 2008 Running on uneven ground: leg adjustment to vertical steps and self-stability. J. Exp. Biol. **211**, 2989-3000. (10.1242/jeb.014357) 18775936

[17] Patla AE, Rietdyk S. 1993 Visual control of limb trajectory over obstacles during locomotion: effect of obstacle height and width. Gait Posture **1**, 45-60. (10.1016/0966-6362(93)90042-Y)

[18] Matthis JS, Barton SL, Fajen BR. 2017 The critical phase for visual control of human walking over complex terrain. Proc. Natl Acad. Sci. USA **114**, E6720-E6729. (10.1073/pnas.1611699114) 28739912 PMC5558990

[19] Domínguez-Zamora FJ, Marigold DS. 2021 Motives driving gaze and walking decisions. Curr. Biol. **31,** 1632-1642. (10.1016/j.cub.2021.01.069) 33600769

[20] Moraes R, Lewis MA, Patla AE. 2004 Strategies and determinants for selection of alternate foot placement during human locomotion: influence of spatial and temporal constraints. Exp. Brain Res. **159**, 1-13. (10.1007/s00221-004-1888-z) 15448958

[21] Patla AE, Armstrong C, Silveira J. 1989 Adaptation of the muscle activation patterns to transitory increase in stride length during treadmill locomotion in humans. Hum. Mov. Sci. **8**, 45-66. (10.1016/0167-9457(89)90023-7)

[22] Gast K, Kram R, Riemer R. 2019 Preferred walking speed on rough terrain: is it all about energetics? J. Exp. Biol. **222**, jeb.185447. (10.1242/jeb.185447) 30910832

[23] Voloshina AS, Kuo AD, Daley MA, Ferris DP. 2013 Biomechanics and energetics of walking on uneven terrain. J. Exp. Biol. **216**, 3963-3970. (10.1242/jeb.081711) 23913951 PMC4236228

[24] Selinger JC, O'Connor SM, Wong JD, Donelan JM. 2015 Humans can continuously optimize energetic cost during walking. Curr. Biol. **25**, 2452-2456. (10.1016/j.cub.2015.08.016) 26365256

[25] Green SJ, Boruff BJ, Bonnell TR, Grueter CC. 2020 Chimpanzees use least-cost routes to out-of-sight goals. Curr. Biol. **30**, 4528-4533.e5. (10.1016/j.cub.2020.08.076) 33007243

[26] Shepard ELC, Wilson RP, Rees WG, Grundy E, Lambertucci SA, Vosper SB. 2013 Energy landscapes shape animal movement ecology. Am. Nat. **182**, 298-312. (10.1086/671257) 23933722

[27] Wilson RP, Quintana F, Hobson VJ. 2012 Construction of energy landscapes can clarify the movement and distribution of foraging animals. Proc. R. Soc. B **279**, 975-980. (10.1098/rspb.2011.1544) PMC3259934 21900327

[28] Nickel BA, Suraci JP, Nisi AC, Wilmers CC. 2021 Energetics and fear of humans constrain the spatial ecology of pumas. Proc. Natl Acad. Sci. USA **118**, e2004592118. (10.1073/pnas.2004592118) 33495339 PMC7865164

[29] Moraes R, Patla AE. 2006 Determinants guiding alternate foot placement selection and the behavioral responses are similar when avoiding a real or a virtual obstacle. Exp. Brain Res. **171**, 497-510. (10.1007/s00221-005-0297-2) 16369789

[30] Matthis JS, Fajen BR. 2013 Humans exploit the biomechanics of bipedal gait during visually guided walking over complex terrain. Proc. R. Soc. B **280**, 20130700. (10.1098/rspb.2013.0700) PMC3673057 23658204

[31] Donelan JM, Kram R, Kuo AD. 2002 Mechanical work for step-to-step transitions is a major determinant of the metabolic cost of human walking. J. Exp. Biol. **205**, 3717-3727. (10.1242/jeb.205.23.3717) 12409498

[32] Nagle F, Balke B, Naughton J. 1965 Gradational step tests for assessing work capacity. J. Appl. Physiol. **20**, 745-748. (10.1152/jappl.1965.20.4.745) 5838725

[33] Ludlow LW, Weyand PG. 2017 Walking economy is predictably determined by speed, grade, and gravitational load. J. Appl. Physiol. **123**, 1288-1302. (10.1152/japplphysiol.00504.2017) 28729390

[34] Mark LS, Jiang Y, King SS, Paasche J. 1999 The impact of visual exploration of judgments of whether a gap is crossable. J. Exp. Psychol. **25**, 287-295. (10.1037//0096-1523.25.1.287) 10069035

[35] Selinger JC, Donelan JM. 2014 Estimating instantaneous energetic cost during non-steady-state gait. J. Appl. Physiol. **117**, 1406-1415. (10.1152/japplphysiol.00445.2014) 25257873

[36] Winter DA. 2005 Biomechanics and motor control of human movement. New York, NY: John Wiley and Sons, Inc.

[37] Rubenson J, Heliams DB, Lloyd DG, Fournier PA. 2004 Gait selection in the ostrich: mechanical and metabolic characteristics of walking and running with and without an aerial phase. Proc. R. Soc. Lond. B **271**, 1091-1099. (10.1098/rspb.2004.2702) PMC1691699 15293864

[38] Minetti AE, Ardigo LP, Reinach E, Saibene F. 1999 The relationship between mechanical work and energy expenditure of locomotion in horses. J. Exp. Biol. **202**, 2329-2338. (10.1242/jeb.202.17.2329) 10441084

[39] Willems PA, Cavagna GA, Heglund NC. 1995 External, internal and total work in human locomotion. J. Exp. Biol. **198**, 379-393. (10.1242/jeb.198.2.379) 7699313

[40] Cavagna GA, Kaneko M. 1977 Mechanical work and efficiency in level walking and running. J. Physiol. **268**, 467-481. (10.1113/jphysiol.1977.sp011866) 874922 PMC1283673

[41] Moraes R, Allard F, Patla AE. 2007 Validating determinants for an alternate foot placement selection algorithm during human locomotion in cluttered terrain. J. Neurophysiol. **98**, 1928-1940. (10.1152/jn.00044.2006) 17686917

[42] Verhulst P-F. 1845 Recherches mathématiques sur la loi d'accroissement de la population [Mathematical researches into the law of population growth increase]. Nouv. Mémoires l'Académie R. des Sci. B.-lett. Bruxelles **18**, 1-39.

[43] Selinger JC, Wong JD, Simha SN, Donelan JM. 2019 How humans initiate energy optimization and converge on their optimal gaits. J. Exp. Biol. **222**, jeb.198234. (10.1242/jeb.198234) 31488623

[44] Sánchez N, Simha SN, Donelan JM, Finley JM. 2021 Using asymmetry to your advantage: learning to acquire and accept external assistance during prolonged split-belt walking. J. Neurophysiol. **125**, 344-357. (10.1152/jn.00416.2020) 33296612 PMC7948143

[45] Poggensee KL, Collins SH. 2021 How adaptation, training, and customization contribute to benefits from exoskeleton assistance. Sci. Robot. **6**, eabf1078. (10.1126/scirobotics.abf1078) 34586837

[46] Darici O, Temeltas H, Kuo AD. 2020 Anticipatory control of momentum for bipedal walking on uneven terrain. Sci. Rep. **10**, 540. (10.1038/s41598-019-57156-6) 31953516 PMC6969077

[47] Daniels KAJ, Burn JF. 2018 A simple model predicts energetically optimised jumping in dogs. J. Exp. Biol. **221**, jeb.167379. (10.1242/jeb.167379) 29530971

[48] Kuo AD. 2002 Energetics of actively powered locomotion using the simplest walking model. J. Biomech. Eng. **124**, 113-120. (10.1115/1.1427703) 11871597

[49] Neptune RR, Kautz SA, Zajac FE. 2001 Contributions of the individual ankle plantar flexors to support, forward progression and swing initiation during walking. J. Biomech. **34**, 1387-1398. (10.1016/S0021-9290(01)00105-1) 11672713

[50] Bledt G, Wensing PM, Kim S. 2017 Policy-regularized model predictive control to stabilize diverse quadrupedal gaits for the MIT cheetah. In IEEE Int. Conf. on Intelligent Robots and Systems*,* Vancouver, Canada, 24–28 September 2017, pp. 4102-4109. (10.1109/IROS.2017.8206268)

[51] Cho B, Kim SW, Shin S, Oh JH, Park HS, Park HW. 2022 Energy-efficient hydraulic pump control for legged robots using model predictive control. IEEE/ASME Trans. Mechatron. **28**, 3-14. (10.1109/TMECH.2022.3190506)

[52] Ding J, Han L, Ge L, Liu Y, Pang J. 2022 Robust locomotion exploiting multiple balance strategies: an observer-based cascaded model predictive control approach. IEEE/ASME Trans. Mechatron. **27**, 2089-2097. (10.1109/TMECH.2022.3173805)

[53] Faraji S, Pouya S, Atkeson CG, Ijspeert AJ. 2014 Versatile and robust 3D walking with a simulated humanoid robot (Atlas): a model predictive control approach. In Proc. IEEE Int. Conf. on Robotics and Automation*,* Hong Kong, China, 31 May–7 June 2014, pp. 1943-1950. (10.1109/ICRA.2014.6907116)

[54] Birn-Jeffery AV, Hubicki CM, Blum Y, Renjewski D, Hurst JW, Daley MA. 2014 Don't break a leg: running birds from quail to ostrich prioritise leg safety and economy on uneven terrain. J. Exp. Biol. **217**, 3786-3796. (10.1242/jeb.102640) 25355848 PMC4213177

[55] Render AC, Kazanski ME, Cusumano JP, Dingwell JB. 2021 Walking humans trade off different task goals to regulate lateral stepping. J. Biomech. **119**, 110314. (10.1016/j.jbiomech.2021.110314) 33667882 PMC8081051

[56] Ruina A, Bertram JEA, Srinivasan M. 2005 A collisional model of the energetic cost of support work qualitatively explains leg sequencing in walking and galloping, pseudo-elastic leg behavior in running and the walk-to-run transition. J. Theor. Biol. **237**, 170-192. (10.1016/j.jtbi.2005.04.004) 15961114

[57] Kuo AD, Donelan JM, Ruina A. 2005 Energetic consequences of walking like an inverted pendulum: step-to-step transitions. Exerc. Sport Sci. Rev. **33**, 88-97. (10.1097/00003677-200504000-00006) 15821430

[58] Wall J, Douglas-Hamilton I, Vollrath F. 2006 Elephants avoid costly mountaineering. Curr. Biol. **16**, 527-529. (10.1016/j.cub.2006.06.049) 16860724

[59] Daniels KAJ, Burn JF. 2023 Human locomotion over obstacles reveals real-time prediction of energy expenditure for optimized decision-making. Figshare. (10.6084/m9.figshare.c.6662929)

